# Supplementary material for: Calendula officinalis—A Great Source of Plant Growth Promoting Endophytic Bacteria (PGPEB) and Biological Control Agents (BCA)
Source: Microorganisms. 2023 Jan 13;11(1):206. doi: 10.3390/microorganisms11010206 (PMC9865722; doi:10.3390/microorganisms11010206)
Supplement: Supplementary file 1 [file microorganisms-11-00206-s001.zip › microorganisms-2150780-supplementary.pdf]

microorganisms

MDPI

Article

Calendula officinalis—A great source of plant growth promoting endophytic bacteria (PGPEB) and biological control agents (BCA)

Polina C. Tsalgatidou [1,2,*], Eirini-Evangelia Thomloudi [1], Kallimachos Nifakos [1,2], Costas Delis [2], Anastasia Venieraki [3,*] and Panagiotis Katinakis [1]

[1] Laboratory of General and Agricultural Microbiology, Agricultural University of Athens, Iera Odos 75, 11855 Athens, Greece
[2] Department of Agriculture, University of the Peloponnese, 24100 Kalamata, Greece
[3] Laboratory of Plant Pathology, Agricultural University of Athens, Iera Odos 75, 11855 Athens, Greece
* Correspondence: polina.tsalgatidou@go.uop.gr (P.C.T.); venieraki@aua.gr (A.V.)

Supplementary Material

Table S1. Molecular identification of endophytic bacteria based on 16S rRNA gene sequence and their NCBI accession numbers.

Strain	Closest species based on 16S rRNA gene sequence (Accession number)	% Identity	GenBank Accession Number
Cal.r.29	*Bacillus velezensis* CBMB205 (NR_116240.1)	99.73	MW267297
Cal.l.33		99.73	MW266119
Cal.r.31.1	*Bacillus mycoides* NBRC 101228 (NR_113990.1)	99.46	MW273495
Cal.r.17		99.46	MW273439
Cal.r.19	*Bacillus subtilis* BCRC 10255 (NR_116017.1)	99.28	MW266538
Cal.r.22		99.64	MW267132
Cal.f.5	*Bacillus proteolyticus* 4275 (MT544821.1)	99.46	MW267743
Cal.r.1	*Bacillus proteolyticus* MCCC 1A00365 (NR_157735.1)	98.58	MW267751
Cal.l.1	*Bacillus proteolyticus* MCCC 1A00365 (NR_157735.1)	99.37	MW267272
Cal.r.7	*Bacillus cereus* ATCC 14579 (NR_074540.1)	99.73	MW267752
Cal.r.27		99.19	MW268768
Cal.r.33	*Bacillus megaterium* ATCC 14581 (NR_116873.1)	99.28	MW266394
Cal.r.28		99.02	MW266126

Cal.f.2		99.37	MW266131
Cal.r.11	*Bacillus halotolerans* DSM 8802 (NR_115063.1)	99.37	MW271042
Cal.l.11		99.55	MW269980
Cal.l.21		99.01	MW273383
Cal.l.30		99.01	MW273438
Cal.l.20		99.28	MW271029
Cal.f.2.1		99.10	MW27292
Cal.f.6.1		99.82	MW268769
Cal.f.6.3		99.19	MW268770
Cal.f.4		99.73	MW273370
Cal.r.20	*Pseudomonas frederiksbergensis* DSM 13022 (NR_117177.1)	98.73	MW286788
Cal.r.30		99.55	MW287260
Cal.r.38.1		99.37	MW287271
Cal.r.21	*Pseudomonas kilonensis* 520-20 (NR_028929.1)	99.64	MW288015
Cal.r.37.1		99.64	MW287272
Cal.r.15		99.64	MW288016
Cal.r.6	*Pseudomonas koreensis* Ps 9-14 (NR_025228.1)	99.28	MW286787
Cal.r.3		99.19	MW286768
Cal.l.6	*Pseudomonas viridiflava* (NR_117825.1)	99.73	MW287322
Cal.f.9		99.73	MW287324
Cal.r.35	*Rhizobium nepotum* 39/7 (NR_117203.1)	99.62	MW286763
Cal.r.8.2	*Stenotrophomonas rhizophila* e-p10 (NR_121739.1)	99.28	MW287326
